# Randomized controlled trial of early endoscopy for upper gastrointestinal bleeding in acute coronary syndrome patients

**DOI:** 10.1038/s41598-022-09911-5

**Published:** 2022-04-06

**Authors:** Chen-Shuan Chung, Chieh-Chang Chen, Kuan-Chih Chen, Yu-Jen Fang, Wen-Feng Hsu, Yen-Nien Chen, Wei-Chuang Tseng, Cheng-Kuan Lin, Tzong-Hsi Lee, Hsiu-Po Wang, Yen-Wen Wu

**Affiliations:** 1 Division of Gastroenterology and Hepatology, Department of Internal Medicine, Far Eastern Memorial Hospital, New Taipei City, Taiwan; 2 College of Medicine, Fu Jen Catholic University, New Taipei City, Taiwan; 3 Department Internal Medicine, National Taiwan University Hospital, National Taiwan University College of Medicine, Yunlin Branch, Yunlin, Taiwan; 4 Department Internal Medicine, National Taiwan University Hospital, National Taiwan University College of Medicine, Taipei, Taiwan; 5 Division of Cardiology, Department of Internal Medicine, Far Eastern Memorial Hospital, No. 21, Section 2, Nanya South Road, Banciao District, New Taipei City, Taiwan

**Keywords:** Cardiology, Diseases, Gastroenterology, Risk factors

## Abstract

Acute upper gastrointestinal bleeding (UGIB) in acute coronary syndrome (ACS) patients are not uncommon, particularly under dual antiplatelet therapy (DAPT). The efficiency and safety of early endoscopy (EE) for UGIB in these patients needs to be elucidated. This multicenter randomized controlled trial randomized recent ACS patients presenting acute UGIB to non-EE and EE groups. All eligible patients received intravenous proton pump inhibitor therapy. Those in EE group underwent therapeutic endoscopy within 24 h after bleeding. The data regarding efficacy and safety of EE were analyzed. It was early terminated because the UGIB rate was lower than expected and interim analysis was done. In total, 43 patients were randomized to non-EE (21 patients) and EE (22 patients) groups. The failure rate of control hemorrhage (intention-to-treat [ITT] 4.55% vs. 23.81%, p < 0.001; per-protocol [PP] 0% vs. 4.55%, p = 0.058) and 3-day rebleeding rate (ITT 4.55% vs. 28.57%, p = 0.033; PP 0% vs. 21.05%, p = 0.027) were lower in EE than non-EE group. The mortality, minor and major complication rates were not different between two groups. Male patients were at higher risk of minor and major complications after EE with OR (95% CI) of 3.50 (1.15–10.63) and 4.25 (1.43–12.63), respectively. In multivariate analysis, EE was associated with lower needs for blood transfusion (HR 0.13, 95% CI 0.02–0.98). Among patients who discontinued DAPT during acute UGIB, a higher risk (OR 5.25, 95% CI 1.21–22.74) of coronary artery stent re-thrombosis within 6 months was noticed. EE for acute UGIB in recent ACS patients has higher rate of bleeding control, lower 3-day rebleeding rate and lower needs for blood transfusion, but more complications in male patients. Further enrollment is mandatory to avoid bias from small sample size (ClinicalTrial.gov Number NCT02618980, registration date 02/12/2015).

## Introduction

Acute upper gastrointestinal bleeding (UGIB) remains challenging with significant morbidity and mortality despite advancements in pharmacological and endoscopic therapy^1^. Pharmacological therapy with proton pump inhibitors (PPIs) and therapeutic endoscopy using various modalities have been shown to significantly reduce re-bleeding, need for surgery and mortality of patients with acute UGIB^1–3^. Additionally, early endoscopy (EE) within 12–24 h, which not only allows early diagnosis and discharge of patients with low risk features, but also enables risk stratification and endoscopic hemostasis with less transfusion requirements and shorter hospital stay, has been recommended for medium-to-high risk patients^1,2,4^. However, endoscopy carries potential risk for complications. Therefore, to evaluate the pros and cons of EE is of paramount importance to treat high risk patients with acute UGIB, particularly for those with poor cardiopulmonary function^5,6^.

Dual antiplatelet therapy (DAPT) are the cornerstone in the management of patients with acute coronary syndrome (ACS)^7,8^. DAPT or other co-prescriptions further increase the major bleeding risk^8,9^. Consequently, serious peptic ulcer disease (PUD) complications occur in a significant proportion of ACS patients^10–12^. However, endoscopy hemostasis in ACS patients may impose risks for cardiopulmonary compromised^13^. Endoscopy within the first week after myocardial infarction (MI) seems to be associated with higher risk for cardiovascular events because of fragile remodeling myocardium, and the safety and timing of endoscopy is not well understood among ACS patients^14,15^. Given the lack of guidelines and randomized control trials (RCTs), gastroenterologists are always reluctant to perform endoscopy in ACS patients due to potential adverse events. In this study, we aimed to evaluate the efficacy and safety of EE versus pharmacological therapy alone for management of acute UGIB in ACS patients.

## Materials and methods

### Study design and randomization

A multicenter RCT of recent ACS patients presenting with acute UGIB was conducted in three tertiary centers (Far Eastern Memorial Hospital, Hsin-Chu Branch and Taipei Branch of National Taiwan University Hospital) in Taiwan. All methods were performed in accordance with the relevant CONSORT 2010 guidelines and regulations (ClinicalTrial.gov Number NCT02618980, registration date 02/12/2015) and was conducted according to Declaration of Helsinki. It was approved by the Research Ethics Review Committee of study institutes (FEMH IRB-103062-F, Hsin-Chu NTUH 105-001-F, Yun-Lin NTUH 201411020RIND). Patients with recent ACS, including unstable angina (UA), ST-elevation MI (STEMI) and non-ST elevation MI (NSTEMI) who presented symptoms of acute UGIB were evaluated for enrollment. The inclusion criteria were as follows: (1) age over 20-year-old, (2) ACS episodes in the past 2 weeks, (3) symptoms of UGIB including hematemesis, coffee ground emesis or tarry stool passage accompanied with a decrease in hemoglobin (Hb) level greater than 2 g/dL from baseline. Patients with any one of the following criteria were excluded: (1) malignancy or other advanced disease with a life expectancy of < 6 months, (2) pregnant or lactating women, (3) history of allergy or severe side effects from PPIs, contrast, and iodine, (4) platelet count < 80 k/μL, or prothrombin time INR > 2.0, (5) decompensated (Child-Turcotte-Pugh score B and C) liver cirrhosis, (6) stage 3–5 chronic kidney disease (CKD) (estimated Ccr < 60 mL/min/1.73 m^2^) using Cockcroft-Gault formula, exclusive of end-stage renal disease under renal replacement therapy^16^. All the authors had access to the study data and had reviewed and approved the final manuscript.

Informed consent was obtained from all eligible patients who were randomly assigned to EE or non-EE management. Patients in both groups received bolus intravenous pantoprazole 40 mg followed by continuous infusion (8 mg/h)^3,17^. In the EE group, patients underwent endoscopy within 24 h after onset of UGIB symptoms. All enrolled patients were monitored in cardiac intensive care unit (ICU). At endoscopy, stigmata of hemorrhage (SRH) were treated by endoscopic therapy in combination of any two of the followings: epinephrine submucosal injection, thermocoagulation, hemoclipping, and argon plasma coagulation. Hemostasis was considered initial successful if bleeding had stopped at endoscopy. Antral-biopsy specimens were obtained to a rapid urease test and histopathological examination for *Helicobacter pylori* (*Hp*) study. Patients assigned to non-EE group received medical treatment with PPIs alone and underwent esophagogastroduodenoscopy 2 weeks after enrollment to evaluate the recent SRH. All the enrolled patients in both groups were kept on oral form PPI for 3 months after 3-day infusional PPI therapy and acute UGIB controlled. Proton pump inhibitors were discontinued once peptic ulcer bleeding was excluded by endoscopy. Decision on discontinuation of DAPT was at the discretion of cardiologists depending on cardiac conditions of each enrolled patient.

### Study endpoints

The primary endpoint was failure of control hemorrhage. The secondary endpoints included complication rate, length of hospital stay, units of blood transfusion, re-bleeding rate, needs for repeated intervention (endoscopic therapy, transarterial embolization (TAE), or surgery) for uncontrollable recurrent bleeding. Blood troponin-T, creatine kinase-MB, Hb, hematocrit (Hct) and complete electrocardiogram (ECG) were checked every 8 h within 24 h after enrollment. APACHE II, Rockall and Blatchford scores at intervention were calculated^18^. Primary care team in the cardiac ICU were blinded to the study protocol. The consulted gastroenterologists gave recommendation for management of UGIB after randomization. The clinical outcomes were recorded by primary care team as routine clinical practice.

#### Definition of failure to control hemorrhage

The time frame for acute bleeding episode was defined as 24 h after enrollment. Clinical failure of control bleeding was defined as: hematemesis or nasogastric tube drainage of significant fresh blood or coffee ground substance (≥ 200 mL) ≥ 2 h, or persistent hypovolemic shock after intervention; or 3 g/dL drop in Hb level (or 9% drop of Hct) within 24 h if no blood transfusion; or a decrease in Hb ≥ 2 g/dL or an increase ≤ 1 g/dL, despite 2 or more units of red blood cells (RBC) component transfusion within 24 h.

#### Definition of clinically significant re-bleeding

Clinically significant recurrent bleeding was defined by the followings: vomiting of fresh blood, fresh blood in the nasogastric tube (NGT) aspirate, hematochezia or melena, and a decrease in Hb ≥ 2 g/dL or an increase less than 1 g/dL, despite 2 or more units of RBC component transfusion after a normal color stool passage, and without coffee ground or fresh blood emesis or in the NGT aspirate.

#### Definition of major and minor complications

Major complications were defined as death and life-threatening arrhythmias within 24 h after randomization. Minor complications were defined as hypotension (< 90/60 mmHg), hypertension (> 180/100 mmHg), tachycardia (> 120 bpm), bradycardia (< 60 bpm), tachypnea (> 24/min.), oxygen desaturation (SpO2 < 90%), and minor arrhythmias.

### Sample size estimation and randomization

The null hypothesis of this study was the superiority of EE over non-EE in the efficacy on bleeding control. The primary efficacy analysis used an intention-to-treat (ITT) approach that included all patients meeting the entry criteria who had completed the follow-up. Approximately 80% of UGIB patients will stop bleeding spontaneously^19^, and rates of hemostasis that resulted from a first endoscopic procedure exceeded 94% in most large studies^20^. However, there was no data demonstrating the outcome of patients under DAPT developing acute UGIB treated medically alone. Therefore, we assumed that about 70% of acute UGIB patients under DAPT would stop bleeding spontaneously without therapeutic endoscopy. As a result, we estimated a sample size of at least 78 patients in EE and non-EE groups in order to achieve a statistical power of 80% at a alpha of 0.05, beta of 0.02 significance level on a two-tailed test, with margin of error of 2% in order to detect a 24% (94% vs*.* 70%) difference. Sealed envelopes with computer generated randomization number (0 for non-EE, 1 for EE group) were used. The time point of randomization was the onset of UGIB symptoms and the non-stratified randomization was performed by gastroenterologists after emergent consultation from ICU. After enrollment, gastroenterologists opened the consecutive envelops for randomization.

### Statistical analysis

Intention-to-treat and per-protocol (PP) analyses were done. Continuous variables were expressed as mean ± standard deviation and the comparisons between two groups were performed using the Student *t* test; categorical variables were summarized as count (%) and the comparisons between groups were made using the χ^2^ or the Fisher’s exact test when appropriate. Univariate and multivariate logistic regression models were performed for evaluation of the risk factors for outcomes in both groups. A two-tailed p value < 0.05 was considered as statistically significant. The statistical analysis was performed using STATA software (version 11.0; Stata Corp, College Station, TX, USA). Interim analysis was performed if there was statistically significant difference in primary endpoint between two groups.

## Results

**Figure 1:**
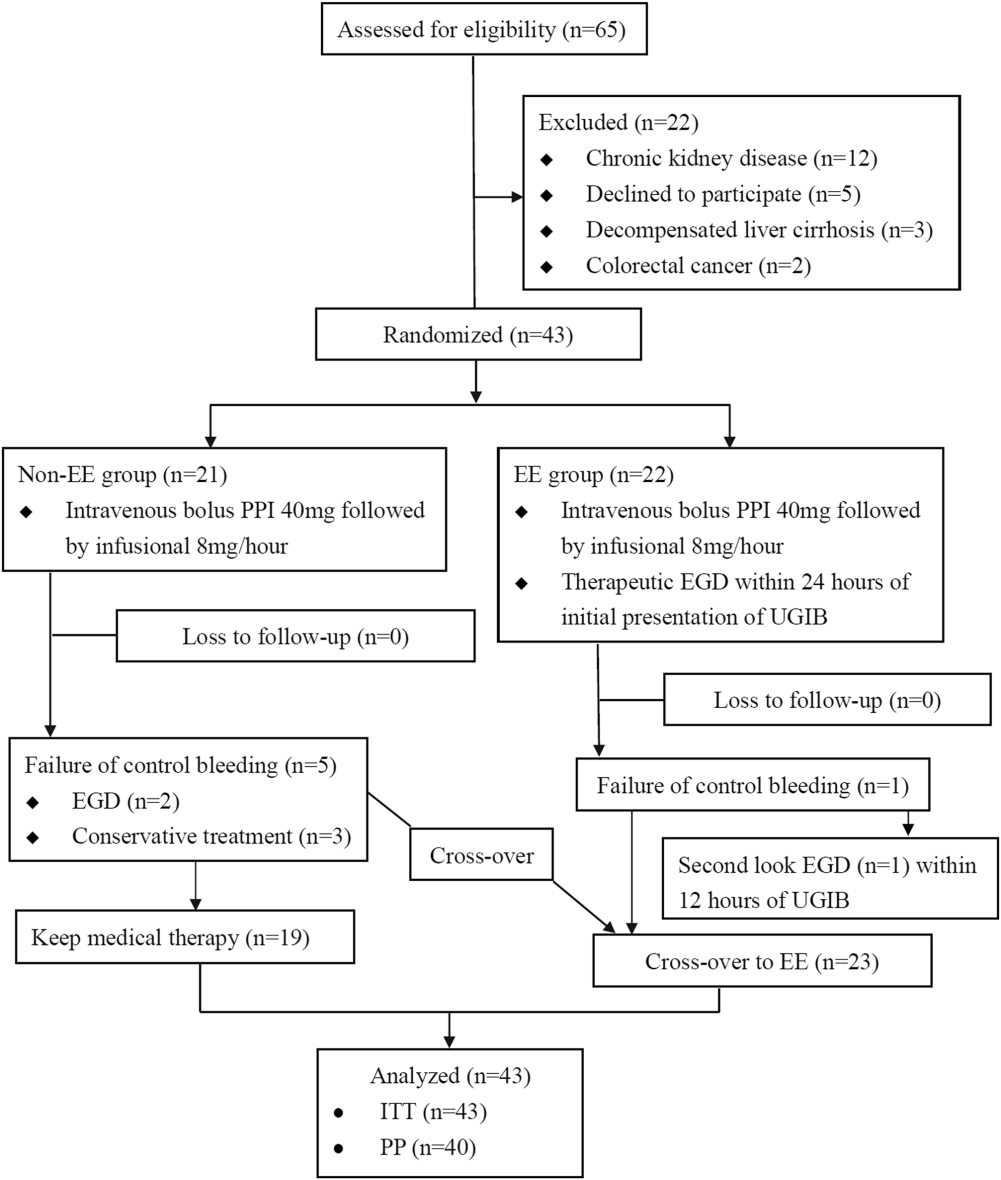
Flow algorithm for enrollment. *EE* emergent endoscopy, *EGD* esophagogastroduodenoscopy, *ITT* intention-to-treat, *PP* per-protocol, *PPI* proton pump inhibitor, *UGIB* upper gastrointestinal bleeding.

Sixty-five patients were assessed for enrollment and 43 (21 in non-EE and 22 in EE group) eligible ACS patients were randomized (Fig. 1 and Table 1). The study was terminated earlier because of slow enrollment. The age (mean ± SD) (70.67 ± 12.82 vs. 63.55 ± 12.19 years old, p = 0.069), gender (female ratio, 61.9% vs. 81.82%, p = 0.146), body mass index (23.71 ± 3.21 vs. 24.29 ± 3.38 kg/m^2^, p = 0.573), status of cigarette smoking (38.1% vs. 59.1%, p = 0.169), history of PUD (19.05% vs. 27.27%, p = 0.523), medications about prophylactic PPI, antiplatelet agents and non-steroid anti-inflammatory drugs (NSAIDs) and timing of UGIB after onset of ACS were not different between two groups. The most common clinical presentation of UGIB in both groups was tarry stool passage. The mean (± SD, range) timing of EE after presentation of acute UGIB was 13.56 (± 6.95, 2.23–22.68) h. The laboratory findings before intervention, cardiac function, proportion of multivessel disease, proportion of patients underwent coronary artery catheterization, and status of discontinuing DAPT were not different statistically between two groups. Patients in EE group had higher Rockall score (5.55 ± 2.70 vs. 4.14 ± 1.35, p = 0.039). Resuming any antiplatelet agent after intervention was numerically earlier in EE than non-EE group (5.13 ± 1.89 vs. 8.63 ± 5.26 days, p = 0.098).

**Table 1:** Demographic data of enrolled patients.

	Non-EE group (n = 21)	EE group (n = 22)	*p*-value
Age (years, mean ± SD)	70.67 ± 12.82	63.55 ± 12.19	0.069
Gender, female/male, n (%)	8 (38.10)/13 (61.90)	4 (18.18)/18 (81.82)	0.146
BMI (kg/m^2^, mean ± SD)	23.71 ± 3.21	24.29 ± 3.38	0.573
Cigarette smoking, n (%)	8 (38.10)	13 (59.10)	0.169
Prior CAD history, n (%)	9 (42.86)	9 (40.91)	0.897
PUD history, n (%)	4 (19.05)	6 (27.27)	0.523
**Drug history, n (%)**			
Prophylactic PPI	13 (61.9)	15 (68.2)	0.666
Aspirin alone	1 (4.76)	2 (9.09)	0.578
Clopidogrel alone	3 (14.29)	4 (18.18)	0.729
Dual antiplatelet	17 (80.95)	16 (72.73)	0.523
NSAIDs	3 (14.29)	2 (9.09)	0.595
MAP (mmHg)	86.44 ± 15.01	84.40 ± 18.77	0.698
**UGIB presentation, n (%)**			
Tarry stool	15 (71.43)	18 (81.82)	0.420
Hematemesis	2 (9.52)	2 (9.09)	0.961
Coffee ground emesis	9 (42.86)	8 (36.36)	0.663
**UGIB severity (mean ± SD)**			
Rockall score	4.14 ± 1.35	5.55 ± 2.70	0.039
Blatchford score	11.14 ± 5.19	11.91 ± 4.47	0.606
APACHE-II	13.24 ± 8.56	17.23 ± 13.50	0.256
Timing of UGIB after onset of ACS (hours, mean ± SD) (range)	45.93 ± 62.26 (3–202)	83.74 ± 96.61 (3–410)	0.137
Timing of endoscopy after UGIB (hours, mean ± SD) (range)	NA	13.56 ± 6.95 (2.23–22.68)	NA
**Laboratory before intervention**			
BUN/CRE (mg/dL, mean ± SD)	39.19 ± 22.69/1.35 ± 2.43	37.91 ± 31.49/0.99 ± 0.29	0.880/0.483
BUN/CRE ratio > 20, n (%)	14 (66.7)	15 (68.2)	0.916
CCr (mL/min, mean ± SD)	69.60 ± 23.70	78.55 ± 32.24	0.308
Hb (g/dL)	9.29 ± 2.68	9.23 ± 3.04	0.943
Platelet count (k/mm^3^)	220.52 ± 70.20	236.18 ± 106.15	0.358
PTINR/aPTT (second)	1.06 ± 0.16/33.56 ± 16.47	1.08 ± 0.12/35.76 ± 26.75	0.554/0.748
**Cardiac function (mean ± SD)**			
CK-MB (ng/mL)	230.15 ± 348.62	225.52 ± 272.95	0.962
Killip score I and II/III and IV, n (%)	10 (47.62)/11 (52.38)	11 (50.00)/11 (50.00)	0.880
TIMI score	6.52 ± 2.84	5.77 ± 2.52	0.364
Multi-vessel disease, n (%)	19 (90.48)	16 (72.73)	0.135
**Management of ACS**			
Medical therapy alone for ACS, n (%)	1 (4.76)	1 (4.55)	
Coronary artery catheterization, n (%)	20 (95.24)	21 (95.45)	0.973
POBA	0 (0.00)	1 (4.76)	0.323
POBAS	16 (80.00)	15 (71.43)	0.558
Without intervention	4 (20.00)	5 (23.81)	0.768
**Discontinuation of antiplatelet, n (%)**			
Aspirin hold	16 (76.19)	15 (68.18)	0.558
Clopidogrel hold	8 (38.10)	9 (40.91)	0.850
All	7 (33.33)	8 (36.36)	0.840
Resuming any antiplatelet agent after intervention, (days, mean ± SD)	8.63 ± 5.26	5.13 ± 1.89	0.098
Follow-up period (years, mean ± SD)	2.52 ± 2.11	2.83 ± 2.44	0.665

*APACHE-II* Acute Physiology and Chronic Health Evaluation-II, *aPTT* activated partial thromboplastin time, *BMI* body mass index, *BUN* blood urea nitrogen, *CAD* coronary artery disease, *CCr* creatinine clearance rate, *CRE* creatinine, *Hb* hemoglobin, *EE* early endoscopy, *MAP* mean arterial pressure, *NSAID* non-steroid anti-inflammatory drug, *POBA* percutaneous old balloon angioplasty, *POBAS* percutaneous old balloon angioplasty with stent, *PPI* proton pump inhibitor, *PTINR* prothrombin time international normalized ratio, *PUD* peptic ulcer disease, *SD* standard deviation, *TIMI* Thrombolysis In Myocardial Infarction, *UGIB* upper gastrointestinal bleeding.

Among five patients in the non-EE group and one patient in the EE group who failed control bleeding in the timeframe of 24 h after onset of UGIB, two of them in non-EE group underwent one time EGD and one in EE group underwent the first EGD after 4 h of the onset of UGIB then the second look EGD after 12 h of the UGIB due to persistent coffee ground substance in NGT with successful hemostasis. The remaining three patients in non-EE group refused endoscopy with PPI therapy. The failure rate of control hemorrhage (ITT/PP, 4.55% vs 23.81%/0% vs. 15.79%, p = 0.0002/0.058) and 3-day rebleeding rate (4.55% vs. 28.57%/0% vs. 21.05%, p = 0.033/0.027) were lower in EE than non-EE group (Table 2) and the trial was stopped due to significant difference in primary endpoint. None of enrolled patient underwent TAE or surgery for UGIB. Regarding the SRH, there was a higher proportion of gastric ulcer in non-EE group (61.90% vs. 36.36%/57.89% vs. 33.33%, p = 0.035/0.053), while *Hp* infection rate (33.33% vs. 40.91%/26.32% vs. 42.86%, p = 0.618/0.273) and length of hospital stay (11.57 ± 5.67 vs. 13.64 ± 10.99 days/11.05 ± 5.66 vs. 13.14 ± 11.01 days, p = 0.446/0.462) were not different between two groups. The units of RBC transfusion were lower after intervention (0.77 ± 1.23 vs. 2.76 ± 2.86 units/0.62 ± 1.02 vs. 2.63 ± 2.99 units, p = 0.005/0.006) in EE than non-EE group. The mortality, minor and major complication rates were not different between two groups, except for a higher proportion of patients with ECG ST-T changes in non-EE group (38.10% vs. 9.09%/41.11% vs. 9.25%, p = 0.024/0.018). One patient died of multiorgan failure due to poor cardiac function within 24 h after enrollment in EE-group.

**Table 2:** Efficacy and safety of early endoscopy versus medical therapy alone for management of acute upper gastrointestinal bleeding in acute coronary syndrome patients.

	Non-EE group		EE group		*p*-value	
	ITT (n = 21)	PP (n = 19)	ITT (n = 22)	PP (n = 21)	ITT	PP
Failure of control bleeding, n (%)	5 (23.81)	3 (15.79)	1 (4.55)	0 (0.00)	0.0002	0.058
**Re-bleeding rate, n (%)**						
3-day	6 (28.57)	4 (21.05)	1 (4.55)	0 (0.00)	0.033	0.027
7-day	2 (9.52)	1 (5.26)	4 (18.18)	3 (14.29)	0.425	0.342
SRH (GU/DU/Others), n (%)	13 (61.90)/7 (33.33)/1 (4.8)	11 (57.89)/7 (36.84)/1 (5.26)	8 (36.36)/8 (36.36)/6 (27.27)	7 (33.33)/8 (38.09)/6 (28.57)	0.035	0.053
*H. pylori* positive, n (%)	7 (33.33)	5 (26.32)	9 (40.91)	9 (42.86)	0.618	0.273
Hospital stay (days, mean ± SD)	11.57 ± 5.67	11.05 ± 5.66	13.64 ± 10.99	13.14 ± 11.01	0.446	0.462
**PRBC transfusion (units, mean ± SD)**						
Before intervention	1.90 ± 1.34	1.89 ± 1.32	2.45 ± 1.63	2.38 ± 1.63	0.234	0.217
After intervention	2.76 ± 2.86	2.63 ± 2.99	0.77 ± 1.23	0.62 ± 1.02	0.005	0.006
Stent re-thrombosis within 6 months, n (%)	6 (28.57)	6 (31.58)	5 (22.73)	5 (23.81)	0.670	0.583
**Complications, n (%)**						
Consciousness change	4 (19.05)	4 (21.05)	2 (9.09)	2 (9.52)	0.358	0.308
Chest pain	8 (38.10)	7 (36.84)	7 (31.82)	7 (33.33)	0.675	0.816
ECG ST-T changes	8 (38.10)	8 (42.11)	2 (9.09)	2 (9.25)	0.024	0.018
Elevating TnT level	14 (66.67)	12 (63.16)	10 (45.45)	9 (42.86)	0.169	0.199
Elevating CK-MB level	0 (0.00)	0 (0.00)	1 (4.55)	1 (4.76)	0.323	0.335
Renal function impairment	16 (76.19)	14 (73.68)	12 (54.55)	11 (52.38)	0.143	0.165
Arrhythmias						
Life threatening	0 (0.00)	0 (0.00)	0 (0.00)	0 (0.00)	NA	NA
Non-life threatening	13 (61.90)	11 (57.89)	16 (72.73)	15 (71.43)	0.461	0.370
Hypotension	6 (28.57)	6 (31.58)	2 (9.09)	2 (9.52)	0.106	0.082
Tachypnea with hypoxemia	4 (19.05)	11 (57.89)	9 (40.91)	15 (71.43)	0.124	0.370
Mortality						
Immediately	0 (0.00)	0 (0.00)	0 (0.00)	0 (0.00)	NA	NA
Within 24 h	0 (0)	0 (0.00)	1 (4.55)	1 (4.76)	0.335	0.335
Index admission	6 (28.57)	5 (26.32)	3 (13.64)	2 (9.52)	0.239	0.163

*CK-MB* creatine kinase-MB, *ECG* electrocardiogram, *EE* early endoscopy, *PRBC* packed red blood cell, *SRH* stigmata of recent hemorrhage, *TnT* troponin-T.

In univariate analysis (Table 3), patients in EE group was not associated with renal function impairment [ITT/PP, hazard ratio (HR) 0.38/0.39, 95% confident interval (CI) 0.10–1.39/0.10–1.49], TnT elevation (HR 0.42/0.44, 95% CI 0.12–1.43/0.12–1.56), arrhythmias (HR 1.64/1.82, 95% CI 0.45–5.94/0.49–6.76), chest pain (HR 0.76/0.86, 95% CI 0.22–2.67/0.23–3.15), discontinuation of DAPT (HR 1.14/1.40, 95% CI 0.33–4.01/0.36–5.49), mortality (HR 0.39/0.29, 95% CI 0.08–1.85/0.05–1.75) and stent re-thrombosis (HR 0.74/0.68, 95% CI 0.19–2.91/0.17–2.73), but lower needs for blood transfusion (HR 0.23/0.23, 95% CI 0.07–0.84/0.06–0.88). In multivariate analysis, less needs for blood transfusion was also noted in EE group (HR 0.13/0.22, 95% CI 0.02–0.98/0.04–1.21) by ITT analysis. Among recent ACS patients with discontinuation of DAPT during acute UGIB, there was a higher risk [odds ratio (OR) 5.25, 95% CI 1.21–22.74)] of coronary artery stent re-thrombosis within 6 months after bleeding episode (Table 4). Male patients were at higher risk of minor and major complications after EE than female ones, with OR (95% CI) of 3.50 (1.15–10.63) and 4.25 (1.43–12.63), respectively.

**Table 3:** Logistic regression analysis to evaluate the outcomes after early endoscopy (adjusted by age, sex, body mass index, smoking status and each parameter).

Variables	Univariate analysis				Multivariate analysis			
	HR (95% CI)		*p-*value		HR (95% CI)		*p-*value	
	ITT	PP	ITT	PP	ITT	PP	ITT	PP
Renal function impairment	0.38 (0.10–1.39)	0.39 (0.10–1.49)	0.142	0.169	3.06 (0.32–29.34)	1.65 (0.25–10.93)	0.333	0.602
TnT elevation	0.42 (0.12–1.43)	0.44 (0.12–1.56)	0.165	0.202	0.46 (0.09–2.36)	0.63 (0.13–2.89)	0.350	0.548
Needs for PRBC transfusion (> 2 units after intervention)	0.23 (0.07–0.84)	0.23 (0.06–0.88)	0.025	0.032	0.13 (0.02–0.98)	0.22 (0.04–1.21)	0.048	0.082
Arrhythmias	1.64 (0.45–5.94)	1.82 (0.49–6.76)	0.451	0.372	1.90 (0.35–10.26)	2.01 (0.42–9.54)	0.459	0.379
Chest pain	0.76 (0.22–2.67)	0.86 (0.23–3.15)	0.666	0.816	0.81 (0.15–4.40)	0.63 (0.14–2.89)	0.811	0.550
Discontinuation of DAPT	1.14 (0.33–4.01)	1.40 (0.36–5.49)	0.835	0.529	1.13 (0.20–6.25)	2.30 (0.37–14.07)	0.892	0.369
Mortality	0.39 (0.08–1.85)	0.29 (0.05–1.75)	0.238	0.178	0.29 (0.03–2.94)	0.26 (0.03–2.23)	0.295	0.220
Stent re-thrombosis	0.74 (0.19–2.91)	0.68 (0.17–2.73)	0.661	0.584	0.24 (0.03–1.92)	2.18 (0.39–12.31)	0.177	0.377

*DAPT* dual antiplatelet agents, *HR* hazard ratio, *PRBC* packed red blood cell, *TnT* troponin-T.

**Table 4:** Risk factors for complications of early endoscopy.

Variables	OR (95% CI)	*p-*value
**Stent re-thrombosis within 6 months**		
Discontinuation of all anti-platelet	5.25 (1.21–22.74)	0.027
**Minor/major complications after EE**		
Age	0.93 (0.83–1.05)/0.62 (0.17–2.30)	0.251/0.474
Male sex	3.50 (1.15–10.63)/4.25 (1.43–12.63)	0.027/0.009
BMI	0.96 (0.70–1.33)/1.05 (0.60–1.89)	0.821/0.873
Timing between EE to UGIB	1.02 (0.99–1.05)/0.85 (0.62–1.15)	0.264/0.284
TIMI score	0.96 (0.62–1.47)/1.64 (0.71–2.37)	0.839/0.243
APACHE-II score	1.05 (0.94–1.18)/1.13 (0.92–1.40)	0.385/0.244
Rockall score	0.92 (0.60–1.41)/1.07 (0.49–2.36)	0.705/0.861
Blatchford score	1.04 (0.82–1.32)/1.66 (0.75–3.68)	0.739/0.209

*APACHE-II* Acute Physiology and Chronic Health Evaluation-II, *BMI* body mass index, *EE* early endoscopy, *TIMI* Thrombolysis In Myocardial Infarction, *UGIB* upper gastrointestinal tract bleeding.

## Discussion

Management of acute UGIB and endoscopic examination in poor cardiac function patients are complex. To our knowledge, this study was the first multicenter RCT to evaluate the efficacy and safety of urgent endoscopy for management of acute UGIB in ACS patients. We have found that EE had higher rate of hemorrhage control, lower 3-day rebleeding rate, and lower needs for blood transfusion than PPI therapy alone. Additionally, early intervention with endoscopy did not increase risk of complications as compared with medical treatment. However, EE should be carefully considered for male patients with recent ACS, while there might be higher complication rate than female patients by logistic regression analysis in our study.

Acute UGIB remains challenging with significant morbidity, particularly composite ischemia (HR 1.94 at 30-day, p < 0.05, and 1.9 at 1-year, p < 0.001), mortality from all-cause (HR 4.87 at 30-day, p < 0.05, and 3.97 at 1-year, p < 0.05) and cardiac events (HR 5.35 at 30-day, p < 0.05, and 3.77 at 1-year, p < 0.05)^1,21–24^. Despite of many benefits from EE, no study has been able to demonstrate that EE leads to a reduction in mortality of acute UGIB. One of the most important reasons is probably due to that mortality of patients with acute UGIB is mainly determined by co-morbidity rather than the success of hemorrhage control^1,2,25^. Moreover, endoscopy carries certain potential risk for complications, including cardiopulmonary events, infectious and thromboembolic events, bleeding, instrumental (perforation, penetration and impaction), and drug reaction from premedication^5,6^. Thus, whether to perform EE in ACS patients presenting acute UGIB is always questionable.

Among ACS patients, DAPT remains cornerstone for the management, especially after coronary artery stenting^7^. Aspirin interferes platelet aggregation activity and has been demonstrated to reduce the risk of cardio-and cerebro-vascular events by as much as 30%, and 18% of all-cause mortality in the secondary prevention of cardiovascular diseases^12,26,27^. However, aspirin increased the risk for GI adverse effects because of inhibition of cyclooxygenase-mediated prostaglandin synthesis. Up to 60% of aspirin users develop GI mucosal lesions under endoscopic examination, especially stomach and duodenum^28^. Clopidogrel, another antiplatelet agent, inhibits platelet function by blocking the adenosine diphosphate receptor on platelets and the CAPRIE trial has shown that long-term clopidogrel monotherapy was more effective and better tolerated than aspirin in reducing combined risk of ischaemic stroke, MI, or vascular death^29^. Clopidogrel seems to be associated with fewer GI adverse effects compared with aspirin^29^. Nonetheless, an animal study revealed that clopidogrel impair the healing of gastric ulcers by suppressing the release of platelet-derived growth factors which are crucial for repair of mucosal defects^30^. Clinical studies have also disclosed that 8–12% of clopidogrel users with a history of PUD bleeding develop recurrent GI bleeding within 12 months^31,32^. A nationwide population-based study demonstrated that the use of clopidogrel increased the risk of UGIB with HR of 3.66 (95% CI 2.96–4.51), especially in elderly, CKD, past history of PUD, and concomitant use of aspirin and NSAIDs^11^. Therefore, how to deal with acute UGIB in the setting of recent ACS is still challenging to gastroenterologists and cardiologists.

In patients with recent ACS, the safety and timing of endoscopy is not well known. Dynamic changes in infarct size may occur since the loss of viable myocardium is progressive after coronary artery occlusion during several hours to days. The infarcted region which itself is a critical determinant of remodeling, incidence of arrhythmias, sudden cardiac death and thus prognosis of ACS, may further expand or contract. Therefore, endoscopy within the first week after MI seems to be associated with higher risk for cardiovascular events. Nonetheless, in several observational cohort and retrospective studies, endoscopy for post-ACS patients has been described as relatively acceptable with complication rates ranging from 7.5 to 48.4%, depending on the definition of complications, timing of endoscopy, and clinical condition of enrolled patients^33–38^. A systemic review of literature has shown that overall complication rate of esophagogastroduodenoscopy after MI was about 1% to 8%, with a large predominance of minor complications^39^. Women seem to experience more periprocedural MI than men (31% vs. 11%, p = 0.058)^36^, and ACS patients who are very ill (APACHE-II score ≥ 16) are more likely to develop endoscopic complications than those with relatively stable condition (21% vs. 2%)^35^. Another retrospective study has also revealed that patients with APACHE II scores > 16 experienced more minor complications (chest pain, abnormal vital signs, or minor arrhythmias) than those with scores ≤ 15 (54.5% vs. 24.2%, p = 0.02)^37^. From the result of a nationwide database involving 1,281,749 ACS patients, endoscopy after coronary arterial catheterization was not associated with a difference in mortality compared with pre-angiogram endoscopy (OR = 0.84, 95% CI 0.60–1.19)^40^. However, design of these studies is mostly retrospective or observational. Given the lack of guidelines and RCTs, gastroenterologists are always reluctant to perform endoscopy for UGIB in ACS patients due to potential risk of complications. In our RCT, we have demonstrated that complication rates were not increased by EE as compared with PPI therapy alone, but higher risk in male patients.

Theoretically, the drug–drug interaction between PPI and clopidogrel reduces the antiplatelet effects and increases the major composite ischemia events. However, several clinical studies and recommendations from international societies suggested prophylactic PPI use for ACS patients taking antiplatelet or antithrombotic agents, particularly those at high risk of UGIB^41,42^. A register-based RCT to examine the effect of screening for risk of UGIB and prophylactic PPI treatment in DAPT patients did not show a reduced incidence of UGIB (1.3% vs. 0.8%, p = 0.38) but a higher compliance with DAPT and reduced risk of recurrent cardiovascular events^40^. In our study, about one-third of patients discontinued DAPT in both groups which was associated with higher risk of coronary artery stent re-thrombosis (Tables 1 and 4). Given that EE could provide initial higher rate of bleeding control, resuming DAPT as early as possible might be achieved to reduce recurrent cardiac ischemic events after coronary artery stenting. According to our results, patients in EE group resumed any antiplatelet agent earlier than those in non-EE group (mean 5.13 vs. 8.63 days), although statistically insignificant (Table 1). Additionally, lower needs for blood transfusion after EE may attenuates complications from over-transfusion of component therapy, particularly in ACS patients who had heart failure and pulmonary edema.

There were some limitations in this study. First, because of more than half ACS patients receiving prophylactic PPI therapy in our institute, the acute UGIB rate was lower than our expectation. Therefore, the number of patients enrolled were smaller than estimated sample size. However, the primary endpoint has been achieved with statistically significance. Secondly, the discontinuing and resuming DAPT was at the discretion of cardiologists rather than a standardized protocol. We discontinued aspirin in the majority (76.19% and 68.18% in non-EE and EE group, respectively) of enrolled subjects because of which is more ulcerogenic than clopidogrel in terms of pharmacological mechanism. It was difficult to suggest the strategy in adjusting DAPT during acute UGIB in ACS patients from our result. Additionally, UGIB status was evaluated only according to clinical symptoms and laboratory data. Among such high-risk patients, UGIB status could be obscured by hemodilution after fluid resuscitation. Finally, we only identify male gender as the significant risk factor for complications from EE in ACS patients. This is probably due to the small sample size and we need further enrollment of eligible patients.

In conclusion, EE for acute UGIB in recent ACS patients has been demonstrated as an efficient and safe procedure for hemorrhage control with lower needs for blood transfusion in this multicenter RCT. Enrollment of more patients and longer study period are warranted to identify risk factors for complications from EE.
